# Supplementary material for: Novel Exosomal miRNA Expression in Irradiated Human Keratinocytes
Source: Int J Mol Sci. 2024 Nov 20;25(22):12477. doi: 10.3390/ijms252212477 (PMC11594671; doi:10.3390/ijms252212477)
Supplement: Supplementary file 1 [file ijms-25-12477-s001.zip › ijms-3285017-supplementary.pdf]

Supplementary Data

Supplementary Figure S1(a)

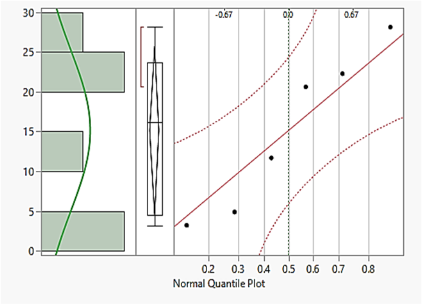

Goodness-of-Fit Test

	W	Prob<W
Shapiro-Wilk	0.92429	0.5368

	A2	Simulated p-Value
Anderson-Darling	0.2698632	0.5912

Supplementary Figure S1(b)

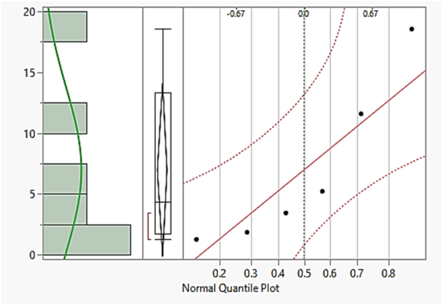

Goodness-of-Fit Test

	W	Prob<W
Shapiro-Wilk	0.8534828	0.1679

	A2	Simulated p-Value
Anderson-Darling	0.4511587	0.1748

Supplementary Figure S1(a): Immediate post-exposure true relative ratio of fluorescence intensity. Data distribution of the immediate post-exposure true relative ratio of fluorescence intensity Supplementary Figure S1(b): true relative ratio of fluorescence intensity 24 hours post-exposure. With a linear relationship. Data distribution of the true relative ratio of fluorescence intensity 24 hours post-exposure. The model displayed a good fit (R-square = 0.967) with a strong positive relationship between the two variables (Correlation=0.983, p-value=0.0004). The model is statistically significant (F (1,4) = 119.72 and p-value = 0.0004). Therefore, the model with a p-value of 0.004 is statistically significant in predicting the true relative ratio of fluorescence intensity based on the equation: True Relative Ratio (within 1hr post-irradiation) = -3.432133 + 0.5305514*Exposure Time
